# Sentiment mining of online comments of sports venues: Consumer satisfaction and its influencing factors

**DOI:** 10.1371/journal.pone.0319476

**Published:** 2025-05-07

**Authors:** Yuxin Zou, Qiuhao Zhao, Bingbing Wang, Guangping Chen

**Affiliations:** 1 School of Physical Education, Shanghai University of Sport, Shanghai, China,; 2 School of Earth Sciences, Zhejiang University, Hangzhou, China; King Caesar University, UGANDA

## Abstract

In the context of consumer economics, it is imperative to consider the functionality of sports venues based on customer demand. However, traditional survey methods are time-consuming, resource-intensive, and coverage-limited. This paper conducted sentiment mining based on Internet big data, deep learning, topic analysis, and social network analysis to capture the satisfaction of consumers and its influencing factors. Findings indicate that activity, courses, and facilities are core factors driving positive comments. Coaches, environment, and activities are key determinants influencing neutral evaluations. Attitude, integrity, and qualifications can trigger negative reviews. The findings offer insights into developing consumer-friendly service for sports venues.

## 1. Introduction

A sedentary lifestyle and lack of exercise pose significant risks to people’s health [1]. Regular physical exercise can effectively reduce health hazards associated with non-communicable diseases such as cardiovascular diseases and obesity [2]. Sports venues, including stadiums, sports halls, clubs, gyms, and fitness venues, play a vital role in promoting sports participation and the development of the economy. They provide spaces for training, competitions, and public enjoyment, accommodating a wide range of sports activities [3]. However, current configuration and limited activity of sports venues do not align with the diverse needs of consumers, leading to an insufficient supply to meet the growing demand for sports [4].

Existing studies have primarily focused on resource allocation [5] and operational utilization [6] from the perspective of venue operators, whereas neglecting the exploration of consumer satisfaction and genuine needs. Satisfaction, as a psychological state, refers to the subjective sentiment generated by the public after receiving a specific service [7,8], involving a comparison between perceived service quality and expectations [9]. By analyzing the consumer satisfaction and its influencing factors, suppliers can make targeted improvement to enhance the service quality [10]. Consequently, it is also crucial to investigate consumers’ needs for sports venues to improve the quality of venue facilities and services. However, existing studies on sport venues lack in-depth analysis of consumer demand and its causing mechanism [11].

Furthermore, the relatively few existing studies on consumer evaluation mainly acquire data using interviews and questionnaires [12]. For instance, Ma et al. used a questionnaire survey to explore the influence of customers’ identification with the team and the service quality of sports venues on their future behavior intention [13]. Brown asked 603 consumers to fill out a questionnaire and found that sports participation and place attachment affect revisiting intentions [14]. However, these approaches are time-consuming, costly, and offer limited scope. [15]. Recently, the advent of social media big data possessed the advantages of the availability, accuracy, and wide coverage [16]. Commenting on social media has become a popular activity for internet users, with 82% of users consulting online reviews before making decisions, surpassing the influence of traditional media [17]. Social media platforms allow users to access venue information before their workouts, and afterwards, they can share their experiences and evaluations of sports venues and fitness equipment [18]. Consequently, online comment data has gained increasing importance in contemporary research [19,20]. Additionally, with the rise of deep learning technologies, especially the development of natural language processing techniques, effective means have been provided for in-depth analysis of the sentiment and causal mechanisms of internet social data [21,22].

To address the shortcomings in existing research, such as insufficient consumer demand surveys and flaws in data methods. This paper utilizes internet big data, sentiment analysis, topic analysis, and social network analysis to conduct an in-depth analysis of consumer satisfaction and its influencing factors. This research aims to offer insights into developing a consumer-friendly environment for sports venues.

The rest of this paper is laid out as follows: Section 2 reviews previous studies on how consumers view sports venues, and how big data techniques are applied in related work. In Section 3, we explain our research approach, which includes analyzing sentiment, topics, and social networks of online comment data. Section 4 presents our findings from sentiment analysis, the main topics in the comments, and why people’s opinions differ. We conclude in Section 5, discussing the main findings, contributions, limitations and ideas for future research.

## 2. Literature review

### 2.1 Consumer satisfaction of sports venues

Users’ evaluations of sports venues can be understood as consumer satisfaction of the venues [12].Previous research has measured a specific group’s satisfaction through survey questionnaires, showing that the construction quality and service quality of the venue are crucial factors that affect consumers’ perception. For example, García-Fernández et al. investigated 56 fitness venues in Spain and concluded that the most important areas for improvement in fitness centers are the condition of equipment and facilities and the layout of venue facilities [23]. Kim stated that the importance of the venue environment is the highest, followed by satisfaction with the event program, and service quality [24]. Yoshida et al. demonstrated that the atmosphere of venue is important for consumers’ mood [25].

Although there has been some research conducted on consumers’ satisfaction of sports venues, there is a lack of systematic literature on diverse types of sports venues. Previous studies have fragmented the role of sports venues, exploring consumers’ evaluation from the perspective of physical exercise only. However, sports venues are not only places for physical exercise [26], but also places for socializing or watching sports for sports enthusiasts [27]. In addition, related works mainly used questionnaire surveys to measure the cognition of specific groups, but traditional survey methods are time-consuming, resource-intensive, and have limited coverage, which may affect the accurate capture of consumers’ perception [28]. In the era of Internet, online comment data provide a new data source that covers a variety of people groups in real time, which may overcome the demerits of traditional data [29]. Gill et al. conducted research using online data and found that compared with traditional methods, higher quality data could be extracted, and the data obtained from the survey could be managed more conveniently [30]. Meanwhile, there were advantages in the speed of data collection and other aspects. Given the accessibility and wide coverage of Internet big data, this article utilizes social media comment data to mine the consumers’ perceptions on sports venues.

### 2.2 Content analysis technique

With the development of Internet, a series of research has emerged that analyzes user-generated-comments such as online review data [31]. Among them, sentiment analysis, topic extraction, and social network analysis are the most popular techniques.

Sentiment analysis is an important information processing technique [32]. This method explores the positive and negative sentiment tendencies of certain content, such as texts or photos [33]. There are two common sentiment analysis methods: sentiment dictionaries and machine learning [34]. Although sentiment dictionaries were widely used early on, their limitations in handling complex texts led to the development of machine learning-based methods. Updating and expanding these dictionaries demand language experts, resulting in significant human resource consumption [35]. Consequently, in 2018, Google introduced the BERT model revolutionized text classification by integrating context semantics, making it highly effective in text analysis [36] and has spurred its widespread adoption among researchers [37]. With the rapid development of technology, sentiment mining has been widely used in exploring consumer perception. For instance, Sun et al. conducted sentiment analysis technology to analyze the cause mechanism of negative evaluation of e-commerce platform in detail [20]. Despite the unique insight sentiment analysis brings, there is a lack of study utilizing sentiment analysis in analyzing consumer satisfaction in sports venues. Given that sentiment analysis brings unique insights, this study aims to investigate online users’ comments on sports stadiums through sentiment analysis based on BERT’s deep learning model. Besides, topic extraction is an effective method to identify the key topics of text contents and has been commonly used in comment data analysis. For example, Shi et al. analyzed comments on Zhang Guowei’s retirement post using the LDA topic model (Latent Dirichlet Allocation), deducing public sentiment on the matter [38]. Zhang et al. discussed the “Sun Yang case” through LDA models and sentiment analysis, exploring hotspots, online opinions, and public attitudes [39]. While topic extraction has brought insights into the mechanism behind the text big data in many other fields, its application in sports venues is insufficient, with limited exploration of consumer perception. This study aims to investigate the influencing factors of consumer satisfaction of sports venues through topic extraction methods.

Social network analysis is a quantitative technique that studies associations between individuals to reveal underlying social relationships. While SNA has been applied successfully in fields like medicine [40] and tourism [41], The feasibility of this method is verified. This paper attempts to use social network analysis to deeply analyze the mechanism of consumer satisfaction in sports venues from a new perspective. By building an association network among consumers, analyzing the connection relationships between nodes, and the overall structure and characteristics of the network. By using machine learning models, such as BERT and LDA, we were able to analyze a large volume of user-generated online reviews to uncover the underlying emotional responses and themes associated with sports venues. We expect to provide targeted recommendations for the operation and management of sports venues. This study contributes to the growing body of literature that recognizes the importance of integrating both emotional and cognitive dimensions into the analysis of consumer behavior.

## 3. Materials and methods

### 3.1 Data collection

**Fig 1:**
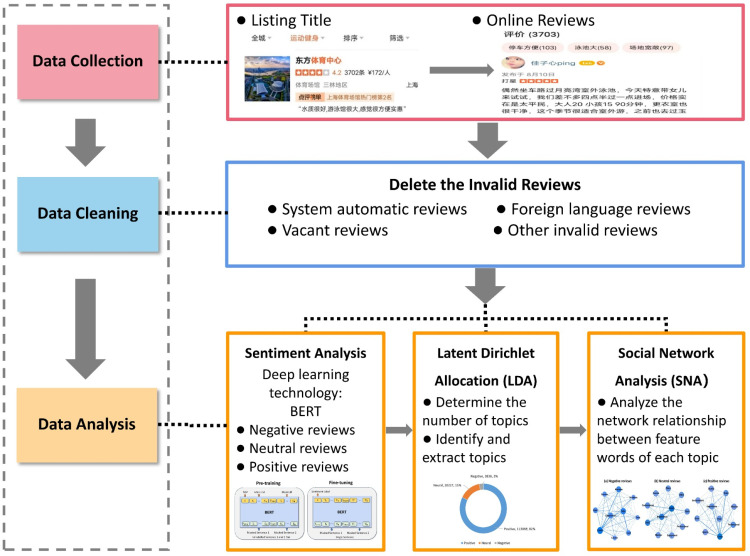
The research processes.

This study employed Python programming to collect reviews of sports venues in Shanghai using API of Dianping. We selected this social media data since Dianping boasts a substantial number of high-quality user reviews in China. A total of 138,313 reviews related to sports venues on Dianping from 2010–2022 were gathered. We followed a thorough multi-step data cleaning process to ensure the quality and relevance of the data set [42]. First, we removed 571 records with missing or null values in key fields such as review content or rating, as these empty records were not useful for analysis. Next, we filtered out 291 reviews that contained irrelevant content, such as advertisements, off-topic remarks, or unrelated information that did not pertain to the sports venues in our study. Finally, as our analysis focuses specifically on Chinese-language reviews, we excluded 319 foreign-language reviews, including those in Korean and English, as they were outside the scope of our research. After completing these steps, the final data set consisted of 137,132 valid reviews. Subsequently, sentiment analysis, LDA modelling, and social network analysis were performed on the cleansed data to explore the sentiment heterogeneity and causing mechanism. The detailed research steps are presented in Fig 1.

### 3.2 Sentiment analysis

This study employs deep learning, specifically Bidirectional Encoder Representations from Transformers (BERT), to analyze the sentiment of review texts. Deep learning, with its ability to automatically extract complex structures from high-dimensional data, has shown exceptional performance in tasks like sentiment analysis [43]. Compared to traditional machine learning models such as logistic regression (LR), support vector machines (SVM), or random forest (RF), BERT offers key advantages. Its bidirectional architecture captures long-range dependencies, enhancing understanding of contextual information [44], while its pre-training and fine-tuning improve generalization across datasets, which is crucial for nuanced sentiment analysis [45]. Numerous studies confirm that BERT outperforms traditional methods like LR, SVM, and RF in sentiment analysis tasks, achieving higher accuracy and F1-scores [46–48]. Therefore, BERT was chosen for its superior performance in handling the complex structures of review texts.

**Fig 2:**
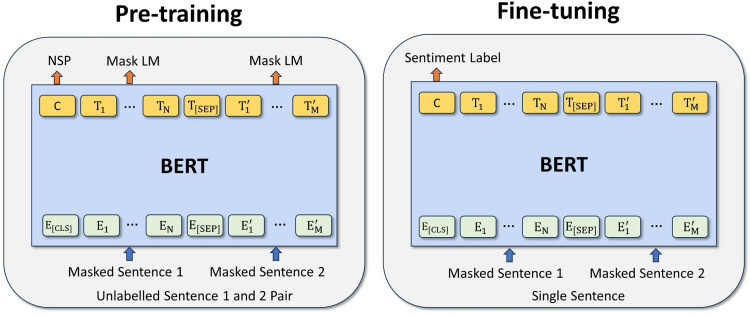
Schematic diagram of the BERT model.

As shown in Fig 2, the input text of BERT is encoded into a low-dimensional representation, and the original sequence input is then approximately reconstructed from this compressed representation. The intermediate feature vector is then capturing key information and features of the reviewer. In this study, the BERT model is fine-tuned using a manually labeled training data set containing 10,000 reviews, categorized into three sentiment classes: positive, neutral, and negative. The labeling process involved a team of five labelers, all of whom were native Chinese speakers with experience in sentiment analysis. To ensure consistency and quality in the labeling, we measured inter-rater reliability using Cronbach’s alpha, which resulted in a value of 0.82, indicating substantial agreement among the labelers. Furthermore, we conducted robustness tests by cross validating a subset of the labeled data with an additional set of reviews to verify the stability of the labels across different reviewers. Any discrepancies were discussed and resolved through consensus among the labelers, ensuring high-quality and consistent labels for the training data set. Subsequently, the sentiment categories of the whole datasets were predicted by the fine-tuned BERT model.

### 3.3 Topic analysis

After conducting sentiment analysis on review data, this study aims to explore the aspects users evaluate sports venues, the most important themes consumers care about, and the reasons behind consumer sentiment by using LDA (Latent Dirichlet Allocation). LDA is an unsupervised machine learning technique used to identify hidden topic information in large document sets or corpora [49]. LDA considers each review as a mixture of various topics, where each topic consists of words representing some “meaning”, such as a concept or aspect [50]. For a given set of online reviews, the topic distribution in each review and the word distribution in each topic can be obtained through the generative process of LDA [51]. Based on the obtained topic distribution and word distribution, the main topics of online reviews can be inferred. LDA has been widely used in the analysis of short text reviews, such as product reviews and online comments, demonstrating its effectiveness in identifying underlying themes even in relatively sparse data [52–54]. Leng et al. explained the sentiment polarity, intensity, and themes of texts related to “naturalized football players” from Sina Weibo using sentiment analysis and keyword frequency analysis [38]. Thus, applying LDA in this study allows for a deeper understanding of the key aspects and themes reflected in user reviews of sports venues.

The fundamental idea of LDA modelling is that each document is constructed by choosing a topic and a word from that topic with a certain probability. Two unknown probability distributions of document-topic and topic-word are the main components of LDA outcomes. The known parameter word-document probability distribution calculates the above two unknown parameters based on the sampling procedure [55]. Formula (1) calculates the probability P for a word to appear in a document. where d, *w*, and *t* stand for the document, word, and subject, respectively; P(w|d) is the likelihood tha*t* a word will occur in the document; P(w|t) denotes the likelihood that a word will appear in the topic; and P(t|d) represents the likelihood that a topic will appear in the document.


P(w|d)=P(w|t)×P(t|d) (1)

### 3.4 Social network analysis

Social network analysis (SNA) is a quantitative technique used for analyzing social networks. Since the 1950s, it has been widely used in sociological research [56]. Social networks can be represented graphically as nodes connected by edges. Its core concept is semantics, defined as the co-occurrence of two words in a sentence. High-frequency words serve as nodes, while the co-occurrence frequency of high-frequency phrases is considered the link between nodes [57]. Based on the LDA results, we constructed an internal co-occurrence matrix for a single topic based on the co-occurrence of specific words and visually presented each topic’s social network construction using GEPHI software. The size of the nodes in the figure is proportional to the frequency of feature words. The bigger the node, the more users pay attention to the feature words. In addition, the thickness of the connection lines between feature words in the figure is proportional to the co-occurrence frequency of feature words on the corresponding nodes. In other words, the thicker the line, the closer the connection between the corresponding feature words.

## 4. Results

### 4.1 Results of sentiment analysis

The BERT model was trained by training datasets and the batch size parameter was adjusted in the range of [4, 8, 16, 32, 64, 128, 256, 512]. It was found that the model evaluation parameters, including precision, recall, and f1-score, achieved the most desirable results when the batch size parameter was set to 256 (Table 1). Then, the optimal BERT model was used to predict the entire dataset.

**Table 1:** Evaluation results for the optimal BERT model’s classification.

Sentiment	Count	Percent	Precision	Recall	F1-score
0 (negative)	3836	2.80%	0.89	0.32	0.47
1 (neutral)	20227	14.75%	0.53	0.53	0.53
2 (positive)	113069	82.45%	0.87	0.91	0.89

**Fig 3:**
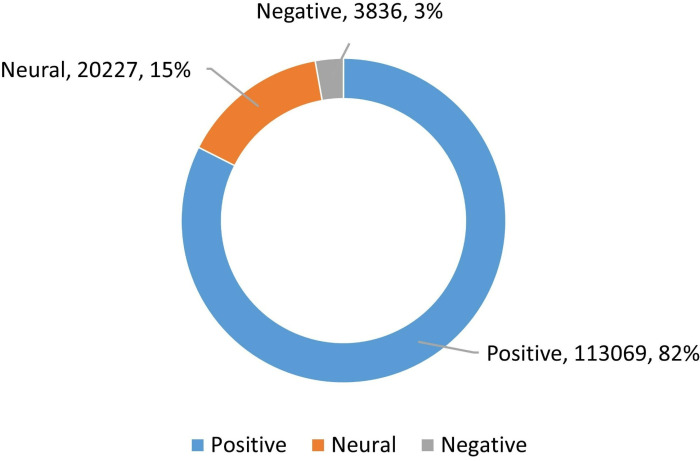
Sentiment analysis result for the review data.

Using the optimal BERT model to analyze the sentiment of all datasets, we obtained 113,069 positive reviews accounting for 82.45%, 20,227 neutral reviews accounting for 14.75%, and 3,836 negative reviews accounting for 2.80% (Fig 3). This indicates that most consumers are satisfied with the sport venues in Shanghai, but there are still areas for improvement.

### 4.2 Results of topic analysis

**Fig 4:**
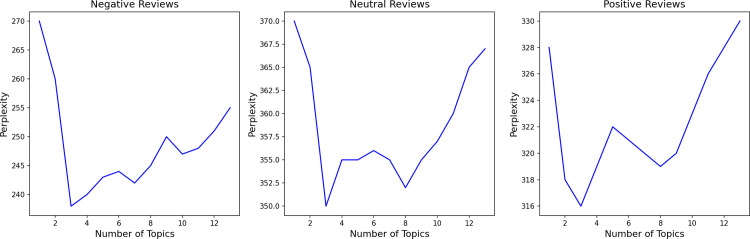
The relationship between perplexity and the number of topics.

To further analyze the mechanism underlying the sentiment, the LDA topic analysis is conducted. Based on the method for determining the optimal number of LDA topics from previous studies [53,58], this study selected three as the best number of topics for negative, neutral, and positive reviews, according to the relationship between perplexity and the number of topics (Fig 4). The results show that for each sentiment category, three topics were identified, with each topic represented by the top 10 feature words (Table 2). Moreover, the environment, feeling, and coaching are the core topics of exercise in sports venues. Thus, these words appear as feature words in multiple topics, exhibiting different meanings across themes.

**Table 2:** Underlying topics in negative, neural, and positive reviews.

Sentiment	Topic	Count	Percent	Keywords									
Negative	Service	1213	31.62%	Attitude	Foreground	Service	Attitude	Staff	Feel	Diathesis	Bad	Range	Experience
(n = 3836)	Course	1032	26.90%	Sale	Coach	Card	Experience	Member	Course	Coaches	Garbage	Price	Feel
	Integrity	1591	41.48%	Manager	Invoice	Brainwashing	Commerce	Certificate	Law	Merchant	Cheat	Swindle	Spend
Neutral	Course	7561	37.38%	Coach	Experience	Feel	Course	Teacher	Profession	Motion	Train	Time	Direction
(n = 20227)	Environment	6533	32.30%	Swimming	Pool	Kid	Environment	Water	Room	Facility	Foreground	Child	Totality
	Activity	6133	30.32%	Concert	Venue	Competition	Place	Stadium	Scene	Traffic	Subway	Events	Park
Positive	Activity	29904	26.45%	Swimming	Experience	Children	Events	Pool	Kid	Friend	Motion	Item	Archery
(n = 113069)	Course	43697	38.65%	Train	Experience	Profession	Motion	Effect	Body	Muscle	Diet	Stature	Strength
	Environment	39468	34.91%	Environment	Facility	Instrument	Feel	Service	Enthusiasm	Equipment	Atmosphere	Room	Equipment

#### 4.2.1 Negative reviews.

Three main themes emerged from the negative reviews of sports venues: service attitude, course expenses, and legal integrity. The primary theme in these negative reviews is the service attitude of the staff, which is a significant concern for users. Service attitude holds great importance across all service industries. In this context, the term “feel” primarily refers to the overall impression left on customers by the staff’s service attitude during workouts at sports venues. Secondly, sports venues were initially established to provide basic exercise spaces for the public. However, as the demand for physical exercise and awareness of the potential harm caused by incorrect fitness practices have increased, value-added services such as fitness courses have gained popularity. Consequently, people pay closer attention to the associated costs. The role of coaches in these fitness courses is crucial. Negative feedback about coaches in user reviews often revolves around whether the course’s value justifies its price.

#### 4.2.2 Neutral reviews.

From the neutral reviews of sports venues, three main themes emerge: private course, environment, and activity programs. The presence of fitness trainers in sports venues, while necessary for some, may not be favored by everyone. Different individuals have varying levels of acceptance for this service, resulting in diverse reviews. Some customers prefer the guidance of a fitness instructor, while others prefer to train independently. Consequently, the existence of this service leads to neutral ratings. The term “feel” in this context refers to both the psychological and physiological impressions that fitness trainers leave on customers during their guidance. Neutral feelings may arise when customers who prefer instructor-led training do not receive sufficient professional guidance. The second major theme in neutral reviews pertains to the environment and facilities of the sports venues. The environment encompasses both tangible and intangible aspects, emphasizing the overall layout and ambiance. Finally, large events held at sports venues, such as sports competitions or concerts, are more likely to elicit neutral emotions from customers. Reviews of these events often consider factors like transportation convenience and accessibility via subway.

#### 4.2.3 Positive reviews.

Based on the positive reviews of sports venues, three main themes emerged: activity programs, training outcomes, and environment. Offering a diverse range of activities is a crucial factor for positive reviews. The overall experience of working out at the sports venues, including perceptions of activity programs and the venue’s ambiance, contributes to positive feedback. Venues that provide a swimming pool tend to receive more positive reviews. The quality of training outcomes is essential, with improved physique, muscle growth, and strength gains reflecting positive feedback. These outcomes are closely linked to professional guidance from instructors and external factors such as diet control. Regarding the environment, the sports atmosphere of the venue is emphasized. However, having basic facilities that meet customer needs is also integral for positive feedback.

### 4.3 Results of social network analysis

#### 4.3.1 Common causes of customer emotional heterogeneity.

This section aims to analyze the factors driving emotional heterogeneity among sports venue customers. We utilized social network analysis to identify commonalities and differences in these factors. To visualize the co-occurrence relationships of specific words within each theme, we created internal co-occurrence matrices. These matrices were then represented as social network structures. In the resulting graphs, the size of the nodes corresponds to the frequency of appearance of the feature words, with larger nodes indicating more user focus on those words. The thickness of the lines connecting feature words represents the co-occurrence frequency between them, indicating the strength of their relationship. Essentially, thicker lines indicate a closer association between the respective feature words. Table 3 presents the number of nodes, edges, average degree, average weighted degree, and graph density for each topic’s network analysis. To enhance clarity, we pruned the network by retaining only the top ten nodes with the highest degree, ensuring that key information is more easily discernible.

**Table 3:** Descriptive statistics of the network analysis.

Sentiment	Topic	Nodes	Edges	Avg. Degree	Avg. Weighted Degree	Graph Density
Negative	Service	2728	101635	37.26	65.89	0.014
	Course	3165	195072	23.22	138.92	0.019
	Integrity	2340	184422	36.14	320.37	0.272
Neutral	Course	3921	243900	62.23	135.62	0.016
	Environment	2884	136032	47.15	96.49	0.016
	Activity	3134	139838	44.62	76.11	0.014
Positive	Activity	2489	119294	47.92	80.67	0.019
	Course	2887	208277	72.14	189.33	0.025
	Environment	2135	144680	62.49	130.09	0.027

**Fig 5:**
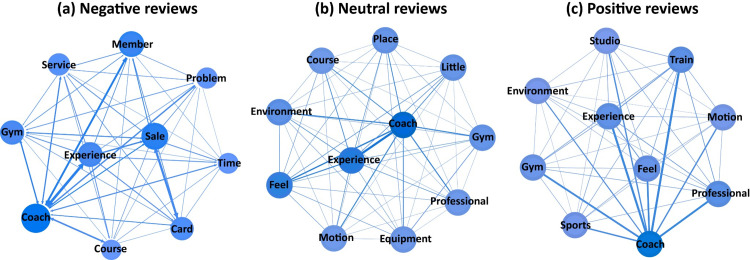
Network diagram of keywords for the course topic.

**Fig 6:**
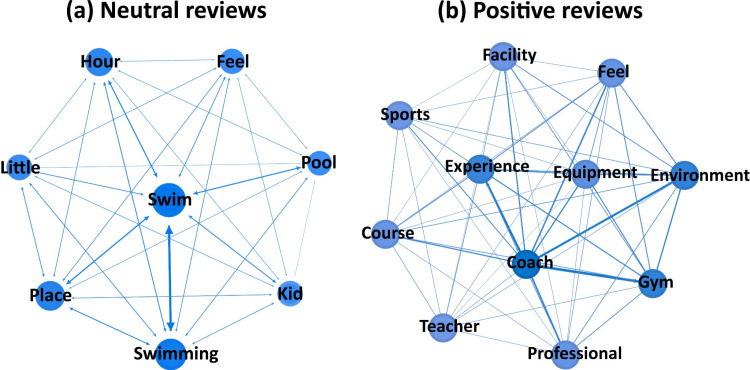
Network diagram of keywords for the environment topic.

**Fig 7:**
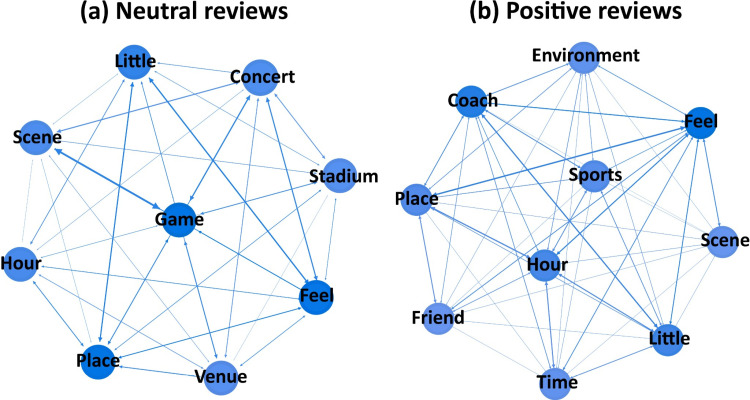
Network diagram of keywords for the activity topic.

(1) Course (Fig 5). Professional guidance is essential when exercising at sports venues. To meet customer demand, some venues offer fitness classes as value-added services. Analyzing terms like “coach”, “member”, and “train” in conjunction with words like “feel”, “experience”, and “service”, suggests that negative reviewers are particularly concerned about the quality of coaches in the paid courses. Neutral reviewers also prioritize the course content, but with a focus on professionalism. The difference lies in the association of cost with value for money. Negative reviews tend to highlight a perceived disparity between the benefits of the courses and the expenses, such as membership fees. Price value refers to the balance between perceived benefits and expenditures. Customers who feel that the benefits do not justify the expenses are more likely to leave negative reviews. This indicates that users have a higher tolerance for course content than for pricing. Even if the exercise content and overall course experience are mediocre, reviews may still be neutral. However, if the price is high and users feel it does not align with the perceived value, their reviews will be more negative.

(2) Environment (Fig 6). While the environment and facilities are crucial aspects of sports venues, high-quality “equipment” can also significantly influence positive emotional reviews from gym-goers, giving sports venues a competitive advantage. The physical “environment”, including layout and facilities, contributes to the overall aesthetic experience. Compared to neutral reviews, a superior environment in the venue tends to evoke more positive emotions from customers. A pleasant ambiance is also essential for positive feedback. The presence of “kid” in sports venues is strongly associated with neutral reviews, suggesting that some fitness enthusiasts pay attention to the inclusion of children in these spaces. Among all sports venues, “swimming” venues are more likely to receive neutral reviews, with water quality being a key concern.

(3) Activity (Fig 7). Whether it is a profit-driven gym, fitness club, or a large non-profit sports arena, a key concern for customers is whether a sports venue provides all the required sports activities and programs. Non-profit sports arenas not only serve as exercise spaces for the public but also host sporting events. The combination of “game” with “feel” and “scene” suggests that when sports venues host sport games or events, the overall experience tends to lead to general reviews. This is because users attend these events for the excitement of the competition rather than solely for fitness, thus the quality of the venue doesn’t evoke strong emotions. Similarly, like neutral reviews, positive feedback is often received for activity programs. These activities are not necessarily large-scale events but rather diverse offerings such as archery and swimming, providing a wider audience with enjoyable experiences. Additionally, the duration of activities is a significant aspect that both neutral and positive reviewers care about.

#### 4.3.2 Individual causes of customer emotional heterogeneity.

**Fig 8:**
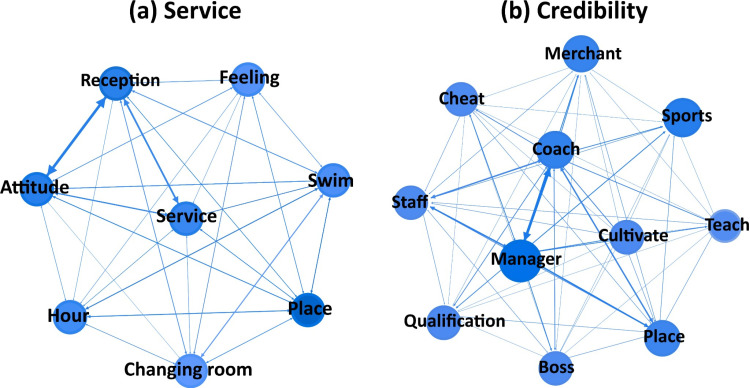
Network diagram of keywords for specific topics.

(1) Service (Fig 8a). As part of the service industry, customer reviews for sports venues mainly focus on whether the services meet customer satisfaction. The combination of terms like “reception” with “attitude” and “service” indicates that a significant proportion of negative reviews for current sports venues stem from poor service attitudes displayed by front desk personnel. However, good service is fundamental to the operation of any venue. Customers expect and consider good service a basic requirement, so they don’t usually leave positive reviews just for that. The perceived attitude of service in sports venues, and the subsequent feelings it induces in customers, is indeed one of the factors influencing negative evaluations. For places like “swimming” pools, the attitude of service, especially in “changing room”, is critically important. This might be due to the increasing number of customers needing to change clothes in swimming venues.
(2) Credibility (Fig 8b). For customers who invest in sports venues, their primary objective is to achieve their workout goals, with price being a secondary consideration. Therefore, what customers are particularly concerned about is the integrity of the venue. They focus on whether the venue can deliver on its promises after making commitments. Within this theme, while customers are wary of being deceived in terms of price, the presence of words like “qualification”, “cheat” indicates that they are equally unwilling to be misled about the effectiveness of their exercises and the credentials of the venue. Despite living in a society governed by the rule of law, instances of lacking integrity still exist. Such unpleasant experiences evoke negative emotions in users.

## 5. Discussion

### 5.1 Main findings

This study utilizes online review data and sentiment analysis to explore consumer satisfaction of sports venues. The sentiment heterogeneity and its influencing factors of comment data are uncovered to improve the service of sports venues.

Research reveals that activities, courses, and facilities are key factors driving positive sentiment. Specifically, in terms of events, consumers prioritize sports venues that offer diverse programs. A rich array of fitness activities not only attracts fitness enthusiasts but is also crucial for attracting parents with children, given the reduced opportunities for play due to lifestyle changes [59]. Urban sports facilities should serve as spaces where children can freely play, catering to various fitness needs. Regarding training outcomes, the fitness community greatly values the tangible changes training brings. They are concerned not just about sweating or muscle soreness but also about the speed of results; hence, sports venues should assign dedicated staff to manage post-training services to improve customer satisfaction. Regarding environmental facilities, upgrades have been shown to enhance consumer perception and significantly boost economic benefits [60]. Furthermore, the venue environment encompasses more than just physical facilities like equipment; it includes service facilities such as well-maintained changing rooms that provide convenience and comfort. Additionally, the atmosphere within the venue is crucial as it enhances positivity, enriches the event experience, and contributes to the overall ambiance [61].

Neutral comments about sports venues are linked to courses, facilities, and activities. Specifically, in private course education, few studies have explored factors from a neutral evaluation perspective. This study suggests that offering private education in sports venues often leads to neutral evaluations because users seek formal and correct exercise methods. However, issues such as lack of professional guidance, inappropriate course arrangements, and unprofessional instructors often result in neutral feedback from customers [62]. Studies have noted problems with private education courses in sports venues, including unmet course demands, lack of professional guidance, and unprofessional instructors. This study also finds that users desire high-quality fitness courses that achieve fitness goals like shaping or slimming. Professional fitness courses and combining networks with apps for effective guidance, including training methods, diet combinations, and sports knowledge, are recommended. Regarding environmental facilities, surveys in Chengdu fitness clubs revealed that customers are concerned about the lack of private space and equipment, affecting satisfaction. Liu and Xiao found that poor environmental cleanliness reduces satisfaction and park use [63], while Wang et al. found that well-maintained facilities in greenways are preferred [64]. Few studies analyze environmental facilities from a neutral perspective. Zhou et al. found that sports facilities impact community sports participants’ satisfaction and willingness to participate [65]. This study found that exercisers are concerned about children’s presence, affecting the overall atmosphere and behavior, such as reluctance to strip in gyms due to children. Children’s crying and playing also impact the environment and others’ fitness experience. Thus, sports venues should enhance the physical environment, cleanliness, and regulate children’s behavior, including banning play and restricting male children from female locker rooms. For competition activities, users are concerned about whether stadiums have adequate facilities and good viewing experience. Large stadiums should provide exercise facilities and support large events, offering a perfect scene and ultimate experience for all guests. Scholars have discussed competition activities in sports venues, including fan experiences, stadium heritage protection, and intelligent lighting systems [66]. Goldberg et al. noted safety issues in large gatherings, highlighting the importance of safety facilities [67]. While customers may not focus on safety features, their absence becomes a critical flaw, leading to neutral evaluations.

Negative reviews of sports venues are related to service attitude, course costs, and legal integrity. Consumers are particularly concerned about service attitude, as it significantly impacts customer experience. Poor service attitudes can severely harm any service industry operator. Studies have shown that the quality of service in fitness venues can significantly affect customer emotions. Research also highlighted the importance of demographic factors such as income, education, and age in guiding facility placement strategies, high-end sports venues tend to attract consumers from higher socio-economic backgrounds who may have different expectations and standards for service quality [68]. Regarding course costs, the price of courses is a key concern. Sports venues serve the public, including children, adolescents, and adults, with varying economic capacities. When users with different spending abilities face the same prices, their evaluations naturally differ. Therefore, it is recommended that sports venues offer fitness courses with experienced coaches while controlling prices and providing discounts for different groups, such as students and senior cards. The transition from free public fitness facilities provided by the Chinese government to paid services has led to dissatisfaction among some consumers. In terms of integrity, users are especially concerned about fair trade, rights protection, and information disclosure. Integrity is crucial in sports, as evidenced by numerous studies. Gardiner et al. analyzed integrity issues in sports from multiple dimensions, highlighting the importance of honesty, transparency, and consistency in building trust [69]. Behavioral integrity, the consistency of words and actions, is essential for trust. When consumers encounter dishonest behavior, such as price fraud or false qualifications, they lose trust in the merchants. An individual’s behavioral integrity is often influenced by the surrounding environment, showing gender differences and situational variations [70]. For example, when sports venues offer promotions like top-up and cash deductions but fail to honor these promises, users feel cheated and distrust the venue, potentially giving up on using its services. Therefore, sports venues must ensure transparency in pricing and consistency in commitments and actions to build consumer trust and promote healthy industry development. This approach also aligns with legal requirements for integrity and helps build a harmonious social sports environment.

### 5.2 Theoretical and practical implications

People’s needs are inherently tied to emotions, as emotions drive the emergence of needs. In addition to exploring people’s subjective demands for sports venues, this paper also delves into their sentiments towards these venues. While subjective feelings were previously gauged through surveys, computer-based sentiment analysis has gained prominence [31]. By extracting user-generated content online and employing machine learning techniques, advancements have been made in various fields such as stock markets, elections, pharmaceuticals, software engineering, and cyberbullying [71,72].

One of the strengths of our study is bridging a gap in existing literature. Machine learning techniques are rarely utilized in the sports domain, and our research confirms their applicability in this context. Another strength is the innovative use of data extracted from social media as a proxy for people’s perceptions and satisfaction with sports venues. Online review data is more time-efficient and cost-effective compared to traditional surveys and interviews that are expensive, time-consuming, and labor-intensive. It also enables broader spatial and temporal coverage and easy replication, potentially becoming a standardized method in the future [33].

In addition, our research has important practical significance for the operation and management of sports venues. Through in-depth analysis, we found that negative reviews are often closely related to key factors such as service attitudes, course consumption, and legal integrity. These negative reviews not only reveal the problems consumers encounter when experiencing the services of sports venues, but also provide directions for the improvement of sports venues. To solve these problems, sports venues can actively improve the service attitude, enhance the service awareness and professional quality of employees; Optimize the course consumption structure, ensure the price transparent and reasonable, to meet the actual needs of consumers; Strengthen the construction of legal integrity, safeguard consumer rights and interests, and establish a good corporate image. Through these targeted improvements, stadiums can create a more consumer-friendly environment and improve the quality of service, thereby attracting more consumers and achieving sustainable development. The three themes of service attitude, course consumption and legal integrity extracted from the negative evaluation of sports venues provide us with valuable feedback and suggestions, which will help promote the continuous progress and improvement of the sports venues industry.

### 5.3 Limitations and future research

While the utilization of big data, machine learning, and social media in this study has provided valuable insights into consumer satisfaction with sports venues, there are several limitations and challenges that need to be acknowledged. Firstly, the reliance on single platforms may not capture the consumer experiences of all sports venues, as some venues may utilize alternative platforms like Meituan or other apps. Secondly, our research solely focused on sports venues in Shanghai. It is promising to explore other cities to gain a more comprehensive understanding of the subject matter. Thirdly, the demographics of the survey sample are constrained by the online review data from social media, which introduces inherent biases. Customers who leave reviews are a subset of the overall customer base and may not be fully representative of the wider population. Consumers who are more likely to leave reviews may have stronger opinions, either positive or negative, and this can skew the data. Furthermore, different people may have different preferences for sports venues. This factor may influence their evaluations, and future research should explore the role of socioeconomic dynamics in shaping consumer sentiment. In addition, research has indicated that female users may prefer aerobic machines to pursue a slim figure, while male users may lean towards anaerobic equipment for a more muscular physique [73]. Elderly people are concerned with fitness activities, training instructors, public restrooms, water dispensers, and covered facilities [74]. Higher-income individuals tend to frequent fitness venues more than their lower-income counterparts in Latin America [75]. Therefore, combining data collection methods is recommended to address survivorship bias in the future research. Using surveys alongside reviews could capture feedback from a more representative sample, including those who typically do not leave reviews. Targeting specific demographics would provide broader insights into customer satisfaction and facility quality, leading to more balanced and generalizable conclusions about consumer behaviour in sports venues.
